# Key for crossing the BBB with nanoparticles: the rational design

**DOI:** 10.3762/bjnano.11.72

**Published:** 2020-06-04

**Authors:** Sonia M Lombardo, Marc Schneider, Akif E Türeli, Nazende Günday Türeli

**Affiliations:** 1 MyBiotech GmbH; Industriestraße 1B, 66802 Überherrn, Germany; 2 Department of Pharmacy, Biopharmaceutics and Pharmaceutical Technology, Saarland University, Campus C4 1, 66123 Saarbrücken, Germany

**Keywords:** gold nanoparticles (AuNPs), blood–brain barrier (BBB), drug delivery, liposomes, nanomedicine, polymeric nanoparticles, solid lipid nanoparticles, superparamagnetic iron oxide nanoparticles (SPIONs)

## Abstract

Central nervous system diseases are a heavy burden on society and health care systems. Hence, the delivery of drugs to the brain has gained more and more interest. The brain is protected by the blood–brain barrier (BBB), a selective barrier formed by the endothelial cells of the cerebral microvessels, which at the same time acts as a bottleneck for drug delivery by preventing the vast majority of drugs to reach the brain. To overcome this obstacle, drugs can be loaded inside nanoparticles that can carry the drug through the BBB. However, not all particles are able to cross the BBB and a multitude of factors needs to be taken into account when developing a carrier system for this purpose. Depending on the chosen pathway to cross the BBB, nanoparticle material, size and surface properties such as functionalization and charge should be tailored to fit the specific route of BBB crossing.

## Introduction

Neurological disorders and brain diseases are real burdens for modern societies and healthcare systems. According to the World Health Organization (WHO), in 2000, brain diseases such as Alzheimer’s disease and other dementias ranked 14th among the causes of death worldwide with approximately 0.8 million deceased [1]. This number more than doubled in the last few years. In 2016, these diseases were responsible for 2.0 million deceased, ranking Alzheimer’s disease 5th. Furthermore, according to the WHO, in 2016 stroke was still the second most frequent cause of death worldwide with approximately 5.8 million deceased. These tremendous numbers will continue to grow due to an aging population, with the rise of life expectancy worldwide. Moreover, there is still a large number of unmet medical needs concerning the treatment of most central nervous system (CNS) diseases, as it is the case for stroke where treatments are limited to brain reperfusion. No treatments are available to recover the brain areas damaged by ischemia [2].

**Figure 1:**
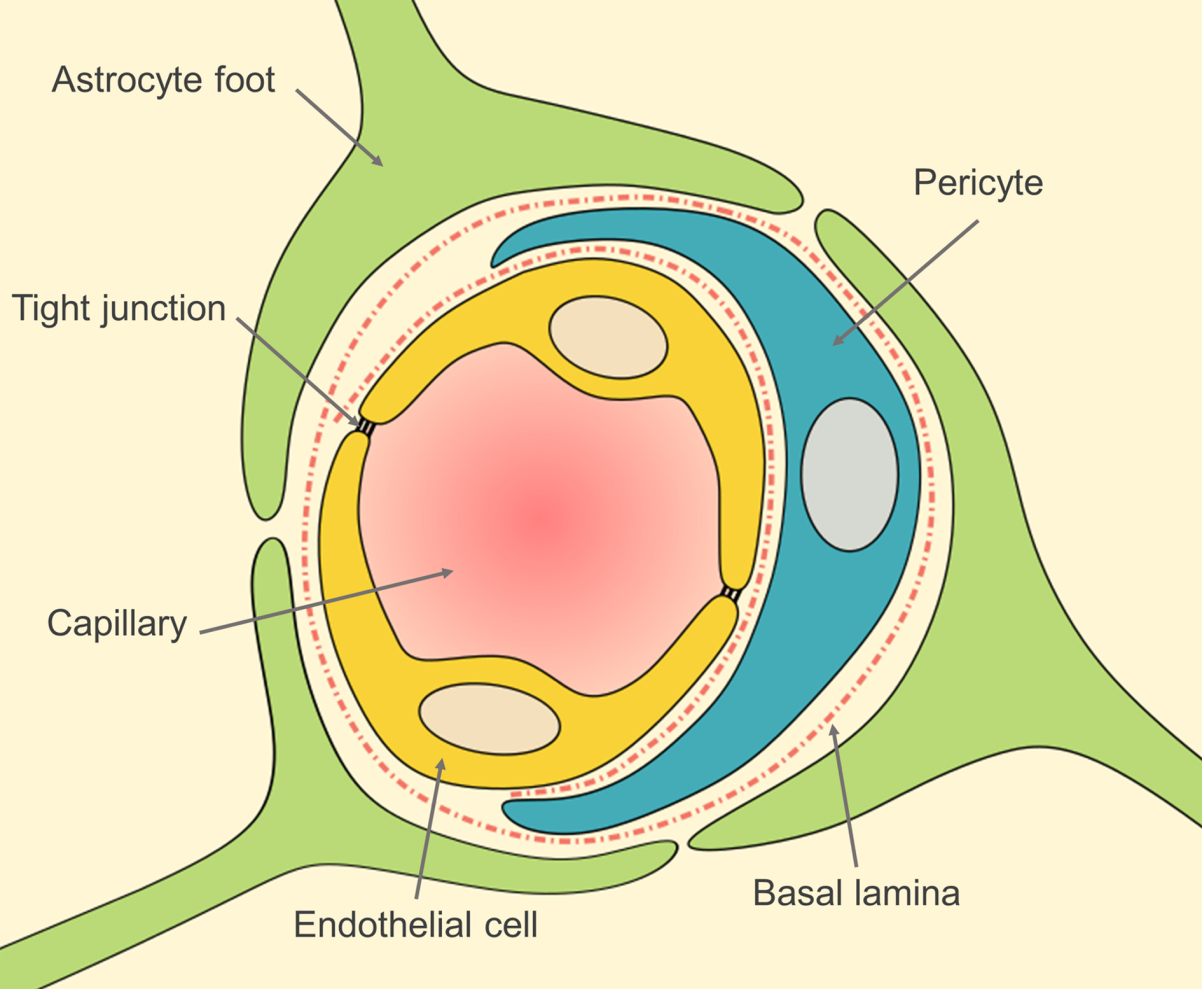
Blood–brain barrier anatomy. Inspired by [5].

One of the main limitations for the treatment of neurological disorders is the difficulty to deliver drugs to the brain. The brain is surrounded by the blood–brain barrier (BBB), a selective barrier formed by the endothelial cells of the cerebral microvessels [3–4]. The surface of the microvessels is the largest interface for blood–brain exchange with an average of 12 to 18 m^2^ in adults [5]. The BBB is responsible for maintaining the brain homeostasis by regulating ion and nutrient transport as well as protecting the brain against neurotoxic molecules [6]. To fulfill its function, the BBB has a unique anatomy. The brain endothelial cells are joined by tight junctions and do not present fenestrations [5–8]. The endothelial cells are surrounded firstly by a discontinuous layer of pericytes and secondly by the basal lamina, adjacent to the astrocyte feet (Figure 1). Unfortunately, most drugs cannot pass the BBB through physiological pathways due to the extreme selectivity of the barrier. This restricts systemic therapeutic treatments for most CNS diseases.

**Figure 2:**
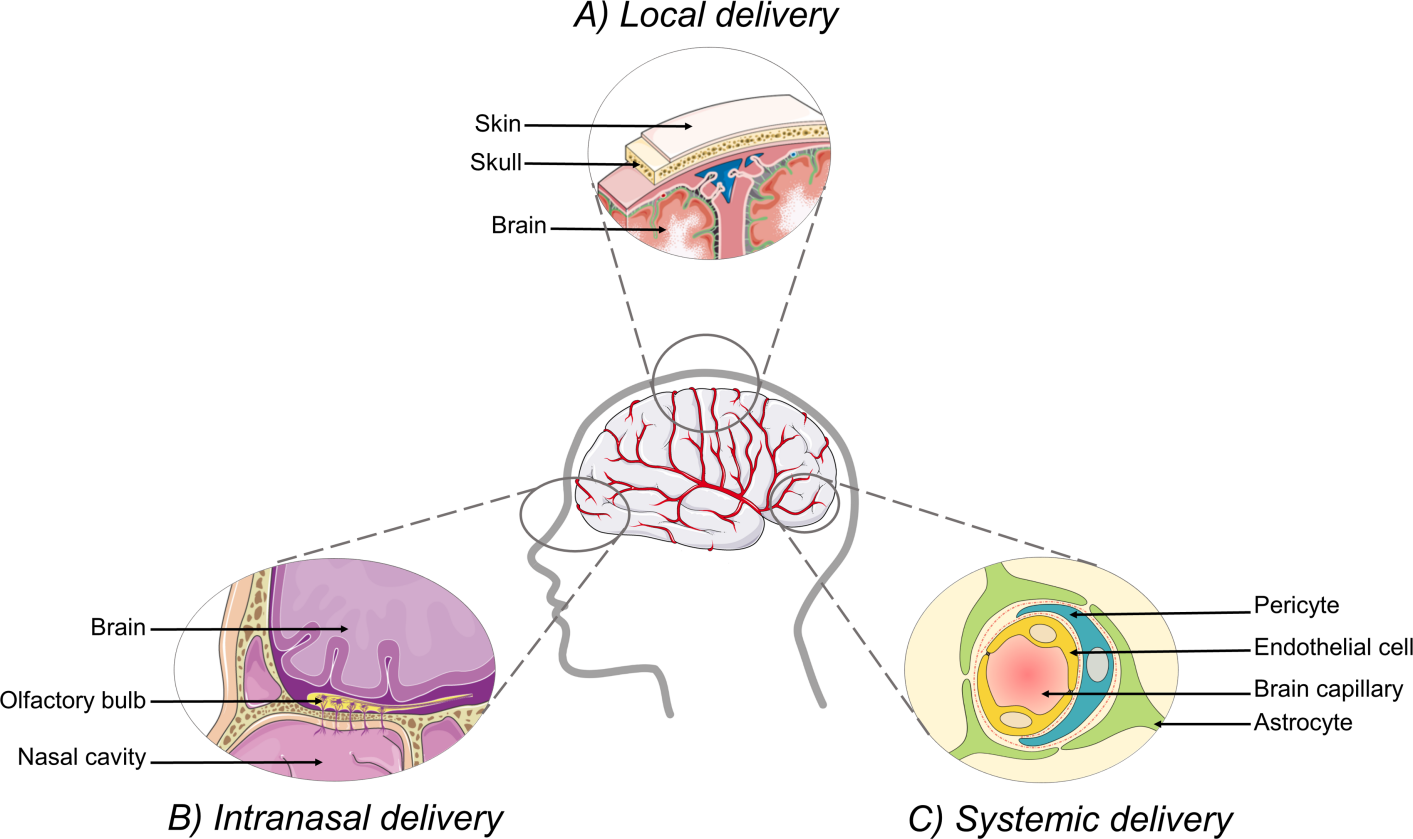
Brain delivery routes. A) Local delivery. Drugs can reach the brain by direct injection through the meninges. B) Intranasal delivery. Drugs can be transported to the brain via the olfactory bulb, located at the top of the nasal cavity. C) Systemic delivery. Drugs can reach the brain by crossing the blood–brain barrier (BBB) around the brain capillaries. Made using cliparts from Servier Medical Art by Servier, https://smart.servier.com/. Original cliparts are licensed under a Creative Commons Attribution 3.0 Unported License, https://creativecommons.org/licenses/by/3.0/.

Different strategies have been suggested to deliver drugs to the brain (Figure 2). First, drugs can be delivered to the brain by local delivery. Local delivery consists of directly delivering the drug to the brain by injection via a catheter or with the help of a convection-enhanced delivery system. Biodegradable polymer implants can also be used for sustained release of the drug [9–10]. These procedures require surgery and are therefore highly invasive. They are mostly used to treat glioblastomas or other brain tumors. Another way to reach the brain by bypassing the BBB is the intranasal route. After reaching the nasal cavity, a drug loaded inside nanocarriers can be transported along the olfactory bulb (olfactory pathway) and the trigeminal nerve (trigeminal pathway) directly to the CNS [11–13]. This innovative route has attracted lots of attention in the last few years and seems promising [14–16]. However, the intranasal route has drawbacks such as a high variability of the delivered dose depending on the state of the nasal mucosa [9,17]. Therefore, despite the difficulty of crossing the BBB, the most popular and well-studied delivery route remains the systemic pathway. One of the classic approaches to increase the ability of drugs to cross the BBB is to modify the molecular structure of the drugs or to use prodrugs. One example of a prodrug is levodopa, a prodrug of dopamine used for the treatment of Parkinson’s disease. However, these options are not always possible depending on the structure of the molecule. Another possibility to increase drug delivery through the BBB is to increase the permeability of the BBB by reversible disruption, either by the use of osmotic agents such as hyperosmolar mannitol injection [18] or physical methods such as ultrasound [19–20]. However, as the BBB is one of the main protection mechanism of the brain against neurotoxins, disrupting it might lead to significant damage to the brain [21].

Finally, an innovative way to solve the permeation problem is to load drugs inside nanoparticles. According to the European Commission, nanomaterials are materials that contain at least 50% of particles in a size range of 1 to 100 nm [22]. More generally, nanoparticles are considered as solid colloidal particles with a size between 1 and 1000 nm [23]. They can be produced from a variety of different materials including polymers, lipids or inorganic materials such gold or iron oxide [21]. The first reported nanoparticles able to pass the BBB were poly(butyl cyanoacrylate) (PBCA) nanoparticles developed by Kreuter et al*.* in 1995. They enabled the successful delivery of the antinociceptive peptide dalargin in vivo [24]. Since then, numerous nanoparticle systems have been studied and optimized for brain delivery of small molecules and peptides [25]. Most of them are polymeric nanoparticles prepared with PBCA and polymers from the poly(ethylene) family such as poly(lactic acid) (PLA) and poly(lactic-*co*-glycolic acid) (PLGA) [25–26]. Liposomes and other lipidic nanoparticles have also been reported as able to pass the BBB [27], as well as protein-based nanoparticles (e.g., human serum albumin) [28], gold nanoparticles [29] and superparamagnetic iron oxide nanoparticles [30].

This review aims to summarize (i) the different pathways to cross the BBB, (ii) the strategies that can be employed to increase nanoparticle BBB permeation without disrupting the BBB, as well as (iii) the different nanoparticle types that can be used for drug delivery across the BBB.

## Review

### Crossing the BBB

**Figure 3:**
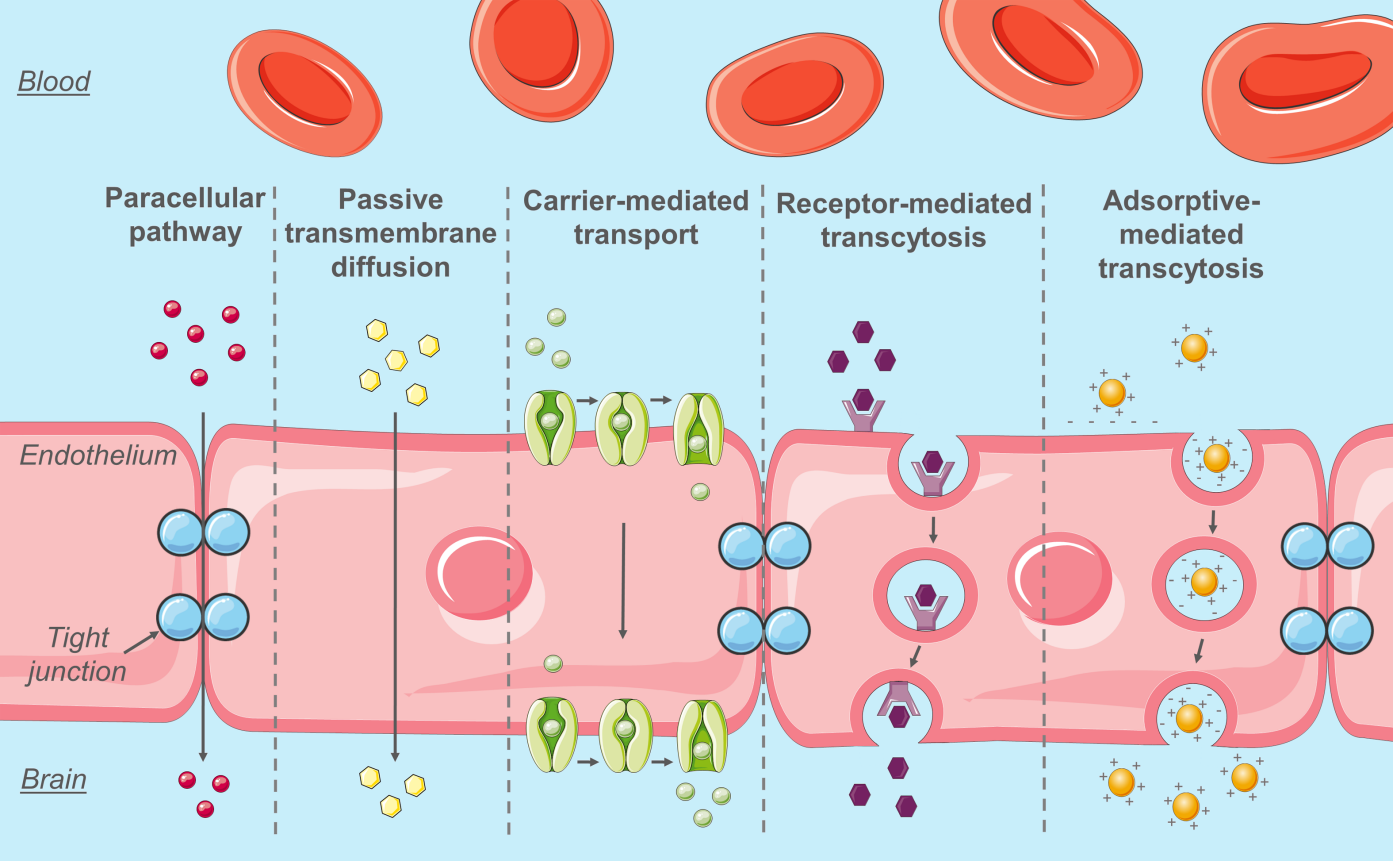
Physiological pathways through the BBB. Inspired by [3]. Made using cliparts from Servier Medical Art by Servier, https://smart.servier.com/. Original cliparts are licensed under a Creative Commons Attribution 3.0 Unported License, https://creativecommons.org/licenses/by/3.0/.

Figure 3 describes multiple pathways to cross the BBB.

#### Paracellular pathway and passive transmembrane diffusion

The tight junctions between the endothelial cells severely limit the paracellular pathway of hydrophilic molecules. Therefore, most molecules have to go through the transcellular pathway to cross the BBB. However, only small lipophilic molecules, with a molecular weight lower than 400 Da and less than eight hydrogen bonds, or small gas molecules (such as CO_2_ or O_2_) can freely diffuse through the BBB by transmembrane diffusion [4]. Furthermore, the BBB endothelial cells have a low degree of pinocytic activity, which again restrains the transport of molecules to the brain [3,8,31].

#### Transport proteins: carrier-mediated transport and efflux proteins

To assure the transport to the brain of specific molecules such as nutrients or amino acids, transport proteins are present on the luminal and basolateral side of the endothelial cells. For instance, GLUT-1, large neutral amino acid transporters (LAT), nucleoside transporters and also organic cation and anion transporters have all been reported to play an important role for sustaining the high metabolic needs of the brain [31–33]. Their substrates can therefore cross the BBB through carrier-mediated transport. These carriers are size- and stereo-selective [34].

ATP-driven drug efflux pumps (ATP-binding cassette (ABC) transporters) also contribute to maintaining the brain homeostasis by excreting possible neurotoxic substances. Active pharmaceutical ingredients (API) can also be substrates of these efflux proteins and therefore be excreted by them. Among the efflux proteins present in the BBB, the most extensively described are P-glycoproteins (P-gp or ABCB1, MDR1 gene product), breast cancer resistance proteins (BCRP/ABCG2) and the multidrug resistance-associated proteins (MRP1, 2, 4 and 5, ABCC) [31,35–38]. With their ability to transport a large variety of compounds, these efflux proteins cause a significant problem for drug delivery.

#### Receptor-mediated transcytosis

Endogenous molecules that do not have a specific transporter can also reach the brain through receptor-mediated transcytosis (RMT). It has been shown that RMT activity in brain endothelial cells is reduced compared to peripheral endothelial cells [39]. However, this pathway remains one of the most promising for drug delivery through the BBB. Transcytosis includes three steps: endocytosis, intracellular vesicular trafficking and exocytosis [40]. Indeed, molecules bind to their receptors on the luminal side of the endothelial cells and endocytosis is initiated. The receptor–ligand complex is invaginated, which leads to the formation of intracellular transport vesicles. The vesicles are then sorted and the ones sorted for exocytosis cross the cell to release the ligand to its basolateral side. The receptor is then recycled [41]. Some of the receptors found on the luminal side of the BBB are transferrin receptor (TfR), insulin and insulin-like growth factor receptor, low-density lipoprotein receptor (LDLR), low-density lipoprotein receptor-related protein 1 and 2 (LRP1 and LRP2), scavenger receptor class B type I (SR-B1), leptin receptor and lactoferrin receptor [34,41]. More recently, nicotinic acetylcholine receptors (nAChRS) and diphtheria toxin receptor have also been described [34,42]. However, some receptors usually found on peripheral endothelial cells are not expressed, e.g., the albumin receptor [40,43].

Three major categories of endocytic vesicles have been identified and described in the brain endothelial cells: clathrin-coated pits, caveolae and macropinocytosis vesicles. Clathrin-coated pits are involved in most of the internalization processes mediated by receptors such as TfR or insulin receptors [39–40]. After endocytosis, the vesicles converge in the early endosome network, which functions as an intracellular sorting station. From there, cargo can be transported via sorting tubules to the basolateral side of the cells for exocytosis. Cargo can also stay in the early endosomes, which can mature in late endosomes and multi-vesicular bodies. Multi-vesicular bodies can either shuttle their cargo for exocytosis or fusion with lysosomes, where cargo degradation can occur. However, the cargo can avoid this fate by being shuttled back from the lysosomes to the Golgi apparatus and early endosomes by retrograde transport [39]. The intracellular pathway taken by the vesicles depends on the receptor, on the internalization pathway (clathrin-mediated or caveolae) and also on the type of ligand binding to the receptors [39]. Furthermore, it has been shown that bEND3 cells (mouse brain microvascular cell line) had less nanoparticles colocalized to the Golgi apparatus and lysosomes than C6 cells (rat glioma cell line), suggesting that intracellular vesicles could avoid lysosomes in brain endothelial cells [43–44]. Finally, the exocytosis process has not been described very well. It is still unclear if the tubules and multi-vesicular bodies merge directly with the basolateral membrane or if they release their cargo in basolateral endosomes that subsequently merge with the basolateral membrane. However, it has been shown that brain endothelial cells generate microvesicles that are released toward the brain [43].

#### Adsorptive-mediated transcytosis

Finally, another potential physiological way to cross the BBB is through adsorptive-mediated transcytosis (AMT). Whereas RMT needs an interaction between a ligand and a receptor, AMT is a non-specific pathway. Therefore, the binding affinity of AMT is low, but its binding ability is high, leading to similar transcytosis efficiency as RMT [45–46]. AMT occurs through electrostatic interaction between a positively charged molecule, protein or peptide and the negatively charged luminal membrane of the brain endothelial cells. This process depends on energy, time and concentration and lasts for a few minutes. It is thus relatively slow compared to carrier-mediated transport [47–48]. The endocytotic process of AMT is mostly mediated by caveolae [40].

### Strategies to enhance nanoparticle BBB permeation

To increase their BBB permeation ability, most nanoparticles are designed to be able to cross the BBB through transcytosis. To reach this goal, their surfaces have to be modified, either non-covalently with a coating or covalently by functionalization.

#### Coating with surfactants

Coating nanoparticles with a surfactant was the first method used to enhance their BBB permeation ability. The first reported nanoparticle system able to cross the BBB in vivo was developed by Kreuter and co-workers [24]. In their study, PBCA nanoparticles coated with polysorbate 80 (PS80) were able to successfully deliver dalargin, an antinociceptive peptide unable to cross the BBB by itself. A significant increase of analgesia was measured, showing that PS80-coated PBCA nanoparticles were able to deliver dalargin through the BBB to the brain. Following this discovery, different surfactants were tested to coat PBCA nanoparticles [49]. Dalargin-loaded PBCA nanoparticles were coated with polysorbate 20, 40, 60 and 80, poloxamer 184, 188, 388, 407 and 908, Brij^®^ 35 and Cremophors^®^ EZ and RH. Only PBCA nanoparticles coated with polysorbates showed a significant analgesic effect, and the highest effect was obtained for PS80-coated nanoparticles. Further studies showed that PS80 did not cause any toxic effects and did not disrupt the BBB at the dose used [50]. At the same time, Lück published in his thesis that apolipoprotein E (ApoE) was adsorbed on the surface of nanoparticles coated with polysorbate 20, 40, 60 or 80 after their incubation in human plasma [51]. However, ApoE was not adsorbed on uncoated nanoparticles or nanoparticles coated with poloxamer 338 and 407, Cremophor^®^ EL or Cremophor^®^ RH40. Building on this work and to study the mechanism behind the transcytosis of PBCA nanoparticles, dalargin-loaded PBCA nanoparticles were coated with apolipoproteins A-II, B, C-II, E and J with or without precoating with PS80 [52] and the antinociceptive effect of dalargin on mice was measured. A significant increase of the antinociceptive effect was observed for nanoparticles coated with apolipoproteins B (ApoB) and E without precoating with PS80, showing that these apolipoproteins increased the BBB crossing of PBCA nanoparticles. Interestingly, the antinociceptive effect of dalargin was even more pronounced for PBCA nanoparticles precoated with PS80 and overcoated with ApoB and ApoE. In the same study, loperamide-loaded PBCA nanoparticles coated with PS80 were injected to ApoE-deficient and control mice. An antinociceptive effect of loperamide could only be observed in control mice, showing that apolipoproteins were involved in the BBB crossing mechanism of PS80-coated PBCA nanoparticles. Thus, it was concluded that PS80-coated nanoparticles could adsorb apolipoproteins selectively in the blood and cross the BBB through RMT by interacting with LDL receptors present on the luminal side of brain endothelial cells.

In another study by Kreuter’s team, PBCA nanoparticles loaded with doxorubicin and coated with either PS80 or poloxamer 188 (P188) could increase the survival of rats implanted with intracranial glioblastoma [53]. Interestingly, P188 coating was also able to increase the BBB permeation ability of PBCA nanoparticles. However, in their first study, P188-coated dalargin-loaded PBCA nanoparticles were not able to increase significantly the antinociceptive effect of dalargin [49]. Thus, it was proposed that the binding of doxorubicin led to an alteration of the nanoparticle surface properties that allowed ApoE and B to be bound. It was then concluded that the BBB permeation ability of nanoparticles was not only dependent of the surfactant coating but also of the nature of the nanoparticle core composition, not only of the polymer, but also of the API [54].

The ability of surfactants to interact with apolipoproteins was confirmed in another study by Petri et al., where the efficacy of PBCA nanoparticles loaded with doxorubicin and coated with either PS80 or P188 for the treatment of rat intracranial glioblastoma was investigated [55]. The results showed that the antitumor effect of doxorubicin-loaded PBCA nanoparticles was significantly enhanced when they were coated with either PS80 or P188. The plasma proteins adsorbed on coated PBCA nanoparticles were investigated by 2D PAGE and the results showed that a considerable amount of apolipoproteins A-I (Apo A-I) were adsorbed on PS80- and P188-coated nanoparticles. No significant differences of the amount of adsorbed apolipoproteins between PS80- and P188-coated nanoparticles could be observed.

Similar results were observed for PLGA nanoparticles stabilized with PS80 or P188. PLGA nanoparticles loaded with either loperamide or doxorubicin were coated with PS80 or P188 and tested in vivo in rodents [56]. In both cases, P188-coated PLGA nanoparticles showed a higher efficacy than PS80-coated nanoparticles, but both formulations were able to cross the BBB and deliver their cargo. On the other hand, in another study, coumarin-6-loaded PLGA nanoparticles coated with either chitosan or PS80 showed a better crossing ability than P188-coated nanoparticles [57]. This result seems to be in accordance with the suggestion from Kreuter et al. that the core of the nanoparticles could influence the surface properties of the nanoparticles and therefore their ability to bind with apolipoproteins in the blood [54]. PS80 coating was also successful for PLA-*b*-PEG nanoparticles [58] but failed for PLA nanoparticles [59]. PLGA-PEG-PLGA nanoparticles loaded with loperamide and coated with either PS80 or P188 were compared [60]. Both formulations could cross the BBB but P188 seemed to permeate to a higher degree than PS80.

In conclusion, PS80 is nowadays the gold standard for increasing the BBB crossing of polymeric particles as it was shown to be able to increase the apolipoprotein–nanoparticle interaction for a wide range of polymers without any toxicity to the BBB. However, alternatives, such as P188, exist. Furthermore, the efficacy of the coating is also influenced by the composition of the nanoparticle core, i.e., the polymer and API in use.

#### Surface functionalization

To be able to cross the BBB by RMT, nanoparticle surfaces can be functionalized with specific ligands. This approach has been well studied and a multitude of different ligands has been tested. Some of the recent advances for this approach are summarized in Table 1.

**Table 1:** Summary of surface functionalization strategies for improved BBB crossing of nanoparticles.^a^

target	functionalization	nanoparticle	size (nm)	zeta potential (mV)	ref.

scavenger receptor class B type 1 (SR-BI)	apolipoprotein A-I	HSA	250–270	−23 to −36	[61]
			225	−35	[62]
		proticles	120–150	10 to 20	[63]
LDL receptor-related protein (LRP1)	apolipoprotein B and E	HSA	220–240	−37 to −40	[62]
			340 ± 8.6	N.A.	[64]
		SLNs	<200	−13	[65]
			119.7 ± 2.5	−54.3 ± 2.1	[66]
	modified apolipoprotein E peptide	liposomes	123 ± 3	−15.2 ± 1.1	[67]
	angiopep-2	PEG-PCL	<100	−3.28 ± 0.75	[68]
		SLNs	111.4	−16.4	[69]
		AuNPs	39.96 ± 0.57	−19.38 ± 0.58	[70]
		AuNRs	118.5	−10.5	[71]
transferrin receptor	transferrin	PLGA	88.8 ± 27.5	−32.5 ± 8.2	[72]
		SLNs	126.4 ± 2.96	3.7 ± 0.5	[73]
		HSA	183 ± 10	−28 ± 8	[74]
		MSN-PLGA	150	−18.1 ± 0.5	[75]
transferrin receptor	transferrin	liposomes	100	30.2	[76]
			124.5 ± 6.4	−5.19 ± 0.45	[77]
		AuNPs	41	N.A.	[78]
		AuNRs	46.7 × 13.7	N.A.	[79]
	anti-transferrin receptor antibody (OX26)	PEG-chitosan	200	18.23 ± 4.06	[80]
		PLGA	166 ± 2	−13 ± 1	[81]
		HSA	168 ± 5	−28 ± 6	[74]
		chitosan	235.7 ± 10.2	22.88 ± 1.78	[82]
		PLA-PEG	121.8 ± 9.9	18.1 ± 1.3	[83]
		liposomes	117 ± 2	N.A.	[84]
			153 ± 11	−7.5 ± 1.2	[85]
	THR peptide	AuNPs	13 ± 1.7	−41 ± 2	[86]
lactoferrin receptor	lactoferrin	PEG-PLGA	90	−24	[87]
			109	N.M	[88]
		procationic liposomes	123–129	−4.3 to −20.2	[89]
		NLC	99.7–103.8	−5.80 to −17.90	[90]
opioid receptor	glycopeptide g7	PLGA	200–300	−8 to −12	[91]
			183 ± 12	16.9 ± 0.4	[92]
			162–211	−6.1 to −15.2	[93]
			195 ± 12	−13.7 ± 0.8	[94]
insulin receptor	insulin	HSA	190 ± 19	−36 ± 6	[28]
		AuNPs	20	N.A.	[95]
	anti-insulin receptor antibody (29B4)	HSA	157 ± 11	−36 ± 4	[28]
receptor for advanced glycation end products	CLPFFD peptide	AuNPs	12 ± 1.7	N.A.	[96]
		AuNRs	50 × 10	25	[97]
diphtheria receptor	CRM197	PLGA	219 ± 11	−14.2 ± 0.5	[94]
thiamine transporter	thiamine	SLNs	67 ± 8.2	N.A.	[98]
glutathione transporter	glutathione	liposomes	95	−8.3	[99]
			127	N.A.	[100]
phage-display technique	TGN peptide	PEG-PLGA	151	−19.59	[101]
	Tet-1 peptide	PLGA	150–200	−20 to −30	[102]
rabies virus target	RVG29	chitosan–pluronic NC	63 ± 32	12.1 ± 0.8	[103]
		HSA	89.3 ± 1.9	−33 ± 0.9	[104]
		AuNRs	117.7 × 50.3	14.2 ± 2.5	[105]
cell-penetrating peptide	TAT	PLA	157 ± 8.9	2.4 ± 0.3	[106]
		liposomes	124.5 ± 6.4	−5.19 ± 0.45	[77]
		AuNPs	21.4 ± 0.9	N.A.	[107]
	penetratin	PEG-PLA	100	−4.42	[108]
	SynB	PEG-GS	194.6 ± 6.4	31.8 ± 3.1	[109]
adsorptive-mediated transport	cationic bovine serum albumin (CBSA)	PEG-PLA	82.1 ± 4.0	−12.19 ± 1.21	[110]
			329 ± 44	−19	[111]
		liposomes	90–165	N.A.	[112]
		SLNs	94.5 ± 1.5	10.3 ± 0.6	[113]
	trimethylated chitosan	PLGA	136.8–146.7	17.7 to 21.0	[114]

^a^AuNPs = gold nanospheres, AuNRs = gold nanorods, GS = gelatin–siloxane, HSA = human serum albumin, MSNs = magnetic silica nanoparticles, NC = nanocarrier, NLC = nanostructured lipid carrier, N.A. = not available, PEG = polyethylene glycol, PLA = poly(lactic acid), PLGA = poly(lactic-*co*-glycolic acid), PCL = polycaprolactone, SLNs = solid lipid nanoparticles.

As described above, coating nanoparticles with apolipoproteins allows nanoparticles to cross the BBB by interacting with LDL receptor-related protein (LRP1) for ApoB and ApoE [115] and with SR-BI for Apo A-I [116]. In the same way, conjugating nanoparticles with angiopep-2, a ligand of LRP1, also enables BBB crossing. Another extensively studied RMT pathway is through transferrin and lactoferrin receptors by conjugating nanoparticles with their respective ligands, transferrin and lactoferrin [72–74,87–88]. However, these nanoparticles have to face competition with the endogenous ligands of these receptors. This problem can be avoided by using antibodies against transferrin receptors, i.e., OX26, which bind to another binding site of the receptor [74,80–83]. However, it has been shown that OX26 is mostly associated with brain capillaries through the brain parenchyma [117]. It was then hypothesized that the high affinity of the antibody for the TfR might prevent its release from the abluminal surface of brain endothelial cells. The detection of OX26 in the cerebrospinal fluid (CSF) and in neurons only in areas of close proximity to the ventricular system also suggest that OX26 might reach the brain through the blood–CSF barrier [118]. Furthermore, some studies have shown that anti-TfR antibodies with high affinity/avidity for the receptor could be sorted toward lysosomal degradation after endocytosis, whereas antibodies with lower affinity/avidity were sorted for transcytosis [119–120]. To the best of our knowledge, the exact intracellular pathway taken by OX26 inside the brain endothelial cells has not been described well yet. Its high affinity for the TfR receptor might induce its entry into lysosomes inside the cells. However, the high number of papers reporting the ability of nanoparticles conjugated with OX26 to reach the brain seems to indicate either that it is not the case, and/or that OX26 is able to shunt the BBB through the blood–CSF barrier.

Other receptors have also been studied such as the opioid, insulin and diphteria receptors or the receptor for advanced glycation end products (RAGE) [28,91–94,96]. It is also possible to cross the BBB by targeting active transporters such as the thiamine or glutathione transporters [98–100].

Some more advanced techniques have also been developed to find new targeting peptides for the BBB, such as the phage-display technique. This technique is an effective method for isolating novel peptides with specific binding properties. DNA sequences are inserted into bacteriophage genes, resulting in the expression of the encoded proteins on their surface. Biopanning is then used to isolate and amplify phages displaying peptides able to interact with the proteins or cell lines of interest [121]. The results of this technique are entirely dependent on the library of DNA sequences used and attention should be given to select tissue-specific peptides [122]. Among others, two interesting peptides have been identified using this technique: TGN and Tet-1. TGN was identified through in vivo phage display [123]. Mice were injected with a library of phages and phages recovered in the brain were amplified, leading to the identification of TGN peptide as an interesting BBB targeting peptide. Tet-1 was identified through its affinity for the trisialoganglioside (GT1b) receptor, the main neuronal target of tetanus toxin [121]. Tet-1 can interact specifically with motor neurons and is capable of retrograde delivery to the neuronal cells [102]. Thus, conjugating nanoparticles with Tet-1 allows them to shunt the BBB by reaching the brain through retrograde axonal transport.

A short peptide derived from rabies virus glycoprotein (RVG), RVG-29, has also been used to increase brain delivery of nanoparticles [103–104]. RVG-29 interacts specifically with the nicotinic acetylcholine receptor (AchR) on neuronal cells [124]. The exact pathway through which RVG-29 reaches the CNS is not fully understood. One hypothesis could be that RVG-29 reaches the CNS by following the rabies virus pathway, i.e., through retrograde axonal transport from motor neuronal cells. It is also believed that RVG-29 could increase BBB crossing through AMT by increasing the positive charge on the nanoparticle surface [125].

Another investigated way to increase BBB permeation is to use cell-penetrating peptides (CPPs). These peptides usually contain between five and 40 amino acids, including a large amount of basic amino-acid residues, resulting in an overall net positive charge [108,126]. CPPs are able to enhance membrane penetration and cell internalization of their conjugated cargo in a large variety of cells, through pathways not always well known [126–127]. In the case of BBB crossing, their positive charge might induce their transcytosis through the AMT pathway. Multiple CPPs have been used to deliver nanoparticles to the brain such as the HIV-1 trans-activating transcriptor (TAT) [77,106–107], penetratin [108] or SynB [109].

Finally, conjugating nanoparticles with positively charged polymers or proteins such as chitosan or cationic bovine serum albumin (CBSA) allows them to cross the BBB by AMT, as described above [110–112,114]. PEG-PLA nanoparticles were labeled with coumarin-6 and conjugated with either CBSA or BSA in a study by Lu and co-workers [110]. Transcytosis assays were performed on a co-culture BBB in vitro model and showed that CBSA-nanoparticles had an apparent permeability (Pe) seven times higher than that of BSA-nanoparticles. Furthermore, a leaching study of coumarin-6 was performed at pH 4.0 and 7.4 and showed that less than 1% of the dye was released from the nanoparticles after 80 h. Thus, coumarin-6 was an accurate probe for the nanoparticle detection. It was therefore possible to conclude that the fluorescence detected in the abluminal compartment during the transcytosis assay was due to the probe inside the nanoparticles, and not due to free coumarin-6. Hence, it seems that CBSA-conjugated nanoparticles are indeed uptaken by the cells and transported to the abluminal side. They do not just adhere on the surface of endothelial cells and release their cargo. However, AMT is a non-specific pathway. Using this route to reach the brain may lead to more adverse effects than RMT as nanoparticles may also accumulate in other organs [128–129].

#### Influence of size and zeta potential

It has been shown that surface functionalization or surface coating are the most important determinants for BBB crossing. For functionalized nanoparticles, size seems to have little impact and nanoparticles in a large size range (from 12 to 340 nm) have been found to cross the BBB (Table 1). Gao and Jiang studied the influence of the size of methotrexate-loaded PS80-coated PBCA nanoparticles on their ability to cross the BBB in vivo [130]. Nanoparticles with sizes from 70 to 345 nm were studied. Between 170 and 345 nm, no impact of nanoparticle size on the brain delivery of methotrexate could be observed. Only 70 nm nanoparticles showed a slight increase in brain delivery. It has been proven that endocytosis is a size-dependent process and that smaller nanoparticles under 100 nm can be endocytosed more easily by cells [131–133]. Moreover, it has been shown that gold nanoparticles under 15 nm were able to cross the BBB without any functionalization, probably through the transmembrane or the paracellular pathway, whereas gold particles bigger than 50 nm were not found in the brain [134–136]. Thus, very small particles may cross the BBB more easily.

Furthermore, after successfully crossing the BBB, size can have an impact on the diffusion of the nanoparticles through the brain extracellular space (ECS). The ECS is a well-connected foam-like structure formed from the interstitial space between neural cells [137–138]. The ECS possesses a large diversity of dimensions down to 40 nm, and up to 700 nm in local expansions also known as “dead spaces" [137,139]. Thus, small nanoparticles can diffuse further in the ECS and therefore deliver their cargo to the brain more efficiently, whereas bigger particles might get stuck in the narrowest parts of the ECS. However, according to Begley’s brain superhighways theory, nanoparticles could be able to diffuse in the brain tissue through the cytoplasm of astrocytes [140]. The long processes of astrocytes can form cytoplasmic bridges in the brain tissue from the BBB to neurons and to the blood–CSF barrier. It was shown that the aquaporines-4 expressed at the end feet of astrocytes allow for the uptake of water and its solutes from the ECS into their cytoplasms. Water can then be released globally into the brain extracellular fluid. Nanoparticles could follow a similar pathway. They could be endocytosed by the astrocytes at their end feet and then diffuse through their cytoplasms in the brain tissue. Nanoparticles could then diffuse faster and with less size restrictions than through the tortuous ECS.

As already described above, the zeta potential can also have an impact on the BBB crossing ability of nanoparticles. Nanoparticles expressing positive charges on their surface can cross the BBB through AMT. However, positively charged nanoparticles have a faster plasma clearance rate, which lowers their residence time in brain microvessels and therefore their brain delivery is reduced [133]. Furthermore, attention should be given to the toxicity of cationic nanoparticles, as they may alter cell membranes during adsorption. For instance, cationic gold nanoparticles have been shown to be 27 times more cytotoxic than their negative counterparts, due to the disruption of the cell membranes [141–143].

#### Pegylation

Pegylation of nanoparticles increases their circulation time by granting them “stealth” properties, thus increasing their residence time in brain microvessels and their brain delivery [144]. Pegylation alone does not allow nanoparticles to cross the BBB, as it has been shown in multiple studies [87,123,145–146]. However, coating nanoparticles with PEG allows them to better diffuse through the ECS [147]. Indeed, an important constituent of the ECS is the extracellular matrix, constituted of proteoglycans, hyaluronan and small proteins that can interact with nanoparticles and drastically hinder their diffusion [137]. By densely coating 40 nm and 100 nm fluorescent polystyrene nanoparticles with PEG, the nanoparticles were able to diffuse through the brain ECS of live mice, thanks to PEG limiting the adhesive interactions between the ECS and the nanoparticles, whereas the uncoated nanoparticles were stuck in the tissue [147]. Interestingly, neither pristine nor pegylated 200 nm nanoparticles could penetrate the brain tissue due to steric hindrance, confirming the importance of nanoparticle size for ECS diffusion described above. Thus, nanoparticles larger than 200 nm are able to cross the BBB but are unable to move on forward and diffuse through the ECS.

### Nanoparticles for drug delivery through the BBB

#### Polymeric nanoparticles

Polymeric nanoparticles are the most extensively studied nanoparticle system for brain delivery. They can be produced from synthetic or natural polymers. Polymeric particles can cross the BBB when coated with surfactants, as described above, or after surface functionalization, as can be seen in Table 1. To be used for brain delivery, the polymeric nanoparticles need to be biodegradable and biocompatible, thus limiting the choice of polymers. As already discussed above, PBCA nanoparticles were the first nanoparticles shown able to cross the BBB [24]. Soon after, PLA and PLGA nanoparticles demonstrated the same abilities [56]. For example, PLGA nanoparticles loaded with loperamide and functionalized with glycopeptide g7 or with a mutated form of diphtheria toxin (CRM197) have been shown to significantly increase the analgesic effect of loperamide in mice [94]. Furthermore, it was shown that CRM197 allowed the carrier system to cross the BBB by RMT as well as by up-regulation of caveolin-1-mediated transport. Investigation on g7-NPs and CRM197-NP tropisms revealed that both formulations reached all brain areas without impacting the BBB integrity and accumulated in interneurons. Both PBCA and PLA/PLGA polymers are biodegradable and biocompatible polymers. However, PLA and PLGA exhibit some advantages over PBCA. They are FDA-approved and have a slower degradation rate than PBCA, allowing for a more sustained delivery [148–149]. Furthermore, a formulation of PLGA nanoparticles loaded with doxorubicin (NanoBB-1-Dox) has been investigated in a phase-I clinical trial for the treatment of glioblastoma multiforme through systemic chemotherapy [150]. Following the good tolerance of the treatment, NanoBB-1-Dox will be investigated in a phase-II study, which might prove the ability of PLGA nanoparticles to cross the BBB in humans. Some other polymers have also been used to develop nanoparticles for brain delivery such as polycaprolactone (PCL) [68] or chitosan [80,82] but to a lesser extent than PBCA and PLA/PLGA nanoparticles. For instance, enhanced accumulation in an in vivo intracranial glioma mice model of PEG-PCL nanoparticles functionalized with angiopep-2 could be observed by real-time fluorescence imaging [68]. Nanoparticles could be observed in the glioma bed and infiltrating margin, showing that nanoparticles functionalized with angiopep-2 could exhibit dual-targeting abilities. Firstly, angiopep-2 allowed the nanoparticles to cross the BBB through RMT by recognition of LRP1 on the BBB and, secondly, angiopep-2 increased the accumulation of nanoparticles in glioma cells thanks to recognition of the LRP1 on the glioma cells surface.

#### Lipid-based nanoparticles

**Liposomes:** Liposomes are well-known and well-studied nanocarrier systems. They are composed of a lipid bilayer surrounding a hydrophilic core. Liposomes can thus be loaded with a wide variety of hydrophilic and lipophilic cargos [151]. Some liposome formulations have already received marketing authorizations, such as Ambisome^®^, Doxil^®^, Myocet^®^ or more recently Onivyde™. However, no liposomal formulations have received approval for clinical use for the treatment of CNS diseases, although some clinical trials are currently ongoing. A phase-I trial of anti-EGFR-immunoliposomes loaded with doxorubicin, still recruiting, might provide soon clinical information on the ratio between the concentration of doxorubicin in the cerebro-spinal fluid and in the peripheral blood after intravenous administration [152].

Liposomes can cross the BBB through RMT. Positively charged liposomes can also reach the brain through AMT [151]. As shown in Table 1, liposomes have already been conjugated with a variety of ligands such as transferrin [76], lactoferrin [89], anti-transferrin receptor antibody [84–85], glutathione [99–100] or CBSA [112]. In a study by Chen et al. the efficacy of the BBB targeting ligands angiopep-2, T7 and peptide-22, which are specific ligands of, respectively, LRP1, transferrin receptor and LDLR were compared. Liposomes were conjugated with each ligand and their cellular uptake by brain capillary endothelial cells (BCECs) was tested [153]. It was shown that the cellular uptake of liposomes functionalized with peptide-22 was significantly higher than that of liposomes conjugated with angiopep-2 or T7. A dual-crossing glioma targeting liposomal drug delivery system was then developed by conjugating peptide-22 and c(RGDfK), a ligand of integrin α_v_β_3_ that showed ability to target glioma cells, to liposomes loaded with doxorubicin. This formulation was tested in vivo on an intracranial glioma-bearing mouse model. The nanocarrier system was able to cross the BBB and to accumulate in glioma cells, thus improving the cytotoxicity effect of doxorubicin.

To cross the BBB and to target glioma cells, liposomes have also been conjugated with cell-penetrating peptides. For example, in a study by Liu et al., a novel dual receptor recognizing the cell-penetrating peptide R8-dGR, which could bind to both integrin α_v_β_3_ and neuropilin-I receptors, was used to functionalize liposomes [154]. This formulation was tested in vitro and in vivo on intracranial glioma-bearing mice. The R8-dGR-conjugated liposomes could cross the bEnd.3 (murine brain endothelial cells) monolayer in vitro and exhibited a significantly higher accumulation in the brain than non-conjugated liposomes, showing the ability of this carrier system to cross the BBB. It was also shown that these particles could accumulate in the glioma cells and significantly increase the survival time of mice when loaded with paclitaxel. Thus, this formulation could be a promising drug delivery system for antitumor therapy.

**Solid lipid nanoparticles:** Solid lipid nanoparticles (SLNs) are particles with a solid lipid core at room and body temperature [27]. SLNs can be prepared with biocompatible lipids, thus leading to low cytotoxicity [155]. Furthermore, SLNs can be prepared using a cost-effective high-pressure homogenization method. This method avoids the use of organic solvents and can be used at large scale, making SLNs interesting for the pharmaceutical industry [156].

Interestingly, plain and stealth SLNs have been shown to cross the BBB, without any functionalization [157–160]. However, stealth SLNs showed better brain delivery of doxorubicin than pristine SLNs. This effect was even more pronounced in SLNs coated with increasing amounts of stealth agents, due to their longer blood circulation time [158]. These experiments showed that no specific targeting molecules or coatings are necessary for SLNs to cross the BBB. These nanoparticles are inherently able to cross the BBB through a pathway that has not been well investigated.

However, it is also possible to increase the amount of drug delivered to the brain by coating and conjugating SLNs. Coating SLNs with PS80 has been proven to be successful for the brain delivery of curcumin [161]. Studies have shown that PS80 allows ApoE adsorption on SLNs in the same way as for PBCA and PLGA nanoparticles [162].

Surface functionalization of SLNs has also been vastly used to improve their brain delivery. For example, SLNs functionalized with ApoE [65–66], angiopep-2 [69], transferrin [73], thiamine [98] and CBSA [113] have shown increased delivery of their cargos to the brain compared to the molecules alone. For instance, in a study by Dal Magro et al., the brain bioavailability in mice of SLNs functionalized with an ApoE-derived peptide was studied depending on the administration route of the nanoparticles. The nanoparticles were administered either by the intraperitoneal, the intravenous or the intratracheal route [66]. Using in vivo fluorescence molecular tomography revealed that the SLNs could be observed in the brain after intravenous and intratracheal administration, showing their ability to cross the BBB. Furthermore, a superior retention of the nanoparticles in the brain was noticed after intratracheal administration compared to the intravenous and the intraperitoneal route, without inducing any acute inflammatory reaction in the lungs. The mechanism behind this increased brain confinement are not well understood. However, the authors concluded that pulmonary administration seemed to be a feasible strategy for brain delivery.

Interestingly, in a study by Peira et al., SLNs have been successfully loaded with superparamagnetic iron oxide and were able to cross the BBB [163]. Thus, SLNs could be potentially used as carriers for CNS MRI contrast agents.

**Nanostructured lipid carriers:** Although most of the reported applications of nanostructured lipid carriers (NLCs) for brain delivery are using the intranasal pathway [164–167], some studies have shown that NLC are also able to deliver drugs to the brain through the BBB. NLC are lipid-based particles composed of a liquid lipid phase inside a solid lipid phase [27]. The liquid lipid phase allows for a better solubilization of APIs and thus for a higher drug loading capacity than that of SLNs [168].

NLCs loaded with itraconazole prepared with PS80 were able to almost double the itraconazole concentration in the brain compared to the drug alone [169]. In another study, arginine–glycine–aspartic acid peptide (RGD)-modified NLCs were used for the delivery of temozolomide (TMZ) and their efficacy was tested on a glioblastoma multiforme mouse model. The RGD-TMZ/NLCs displayed high antitumor efficacy in vivo, with an inhibition of the tumor four times higher than that of the drug alone [170]. Moreover, in a study by Tsai et al., baicalein was loaded inside NLCs prepared with poloxamer 188. The NLCs revealed 7.5- and 4.7-fold higher baicalein accumulations in the cerebral cortex and brainstem, respectively, compared to the aqueous solution [171]. It is also possible to increase the BBB targeting ability of NLCs by adsorbing lactoferrin at their surface [90]. In a study by Meng et al., results have shown that lactoferrin-modified NLCs were able to accumulate in the brain more than twice as much as pristine NLCs in an Alzheimer’s disease rat model [90].

Thus, even if the BBB permeation potential of NLCs has only been studied to a small extent [172], it seems that this nanoparticle system might be promising for brain delivery through the systemic pathway.

#### Inorganic nanoparticles

**Gold nanoparticles:** Gold nanoparticles are inorganic particles the shape (spheres, shells, rods) and size of which can be tailored depending on the synthesis process [173]. Most studies on the brain delivery of gold nanoparticles used gold nanospheres (AuNPs). AuNPs are composed of a gold core and covalently or non-covalently attached surface ligands. AuNPs have many very interesting properties. They can be easily synthetized and coated or conjugated to carry various cargos, from small molecules to proteins and nucleic acids [174]. Furthermore, AuNPs show low immunogenicity and toxicity due to their inert core [78]. Finally, AuNPs could be used as X-ray contrast agent if their concentration in the brain is sufficiently high to allow for their detection, making them potential carriers for theranostic treatments [174].

It has been shown that the organ distribution of gold nanoparticles depends on the size of the particles. In multiple in vivo studies on rodents, low amounts of AuNPs of 10–15 nm were able to cross the BBB and reach the brain. However, the vast majority of the administered dose was found in the liver and in the blood [134–136]. Moreover, the amount of gold found in the brain was dependent on the AuNP dose, showing no sign of saturation at the doses tested. This suggests that the AuNPs might cross the BBB through a non-saturable pathway such as passive transmembrane diffusion or the paracellular pathway [175]. Of course, depending on the surface ligands of the AuNPs, the pathway taken might differ. Hydrophilic ligands might hinder the transmembrane diffusion of the particles. Surface modifications with specific ligands such as transferrin [78], THR peptide (a transferrin receptor ligand) [86], angiopep-2 [70], insulin [95] and CLPFFD peptide, a peptide derived from β-amyloids able to interact with RAGE [96], have been shown to increase the BBB delivery of AuNPs by allowing them to cross the BBB through RMT. Moreover, AuNPs have been extensively used to reach brain tumors. Due to their small size, AuNPs can diffuse more easily through the disrupted BBB of the brain tumor vasculature, making them a useful carrier for drugs or imaging agents. The use of targeting ligands, such as angiopep-2, TAT or EGF, allows their accumulation to be increased in these specific areas [70,78,107,176].

Although to a lower extent, gold nanorods (AuNRs) have also been used for brain delivery. AuNRs, like AuNPs, exhibit an optical feature called surface plasmon resonance, which allows them to strongly absorb light in the infrared region [177–178]. The advantage of AuNRs over AuNPs is that their aspect ratio (length divided by width) allows for the adjustment of the absorption wavelength in the near infrared (NIR) region (650–1350 nm), thus exploiting the so-called optical window. Light in this wavelength range can penetrate more deeply into the human body thanks to the low absorption by tissue and blood, making these nanoparticles interesting for theranostic treatments [177,179–180]. By excitation of surface plasmon oscillations, local heating can be generated, making these particles interesting for tumor treatment by photothermal therapy [178]. Furthermore, nanorods can be internalized more easily by cells as their increased surface allows them to interact more easily with receptors on the cell membranes [181]. AuNRs have been functionalized to increase their BBB delivery of angiopep-2 [71], RVG29 [105], CLPFFD [97] or transferrin [79]. In a study by Praça et al., AuNPs and AuNRs were functionalized with different amounts of transferrin and tested in vitro on a BBB model and in vivo on mice after intravenous administration [79]. In vitro and in vivo results showed that AuNPs and AuNRs with a medium density of transferrin had the highest transport efficiency across the BBB, due to their lower avidity for the transferrin receptor. The latter allowed them to more easily escape the endosomes to reach the cytosol, thus avoiding the lysosomal pathway. In vivo studies confirmed the ability of functionalized AuNPs and AuNRs to cross the BBB as both formulations were found in the brain of mice in larger quantity than the bare formulations. Interestingly, AuNRs accumulated in the subventricular zone (SVZ), a neurogenic niche, while AuNPs were mostly found in non-neurogenic regions. Disruption of the BBB by NIR light irradiation of AuNRs increased the AuNRs ability to accumulate in the SVZ, making this formulation interesting for targeting neural stem cells.

**Superparamagnetic iron oxide nanoparticles:** Superparamagnetic iron oxide nanoparticles (SPIONs) are based on magnetite (Fe_3_O_4_) or maghemite (γ-Fe_2_O_3_) molecules encapsulated in polysaccharides, synthetic polymers or monomer coatings and have a size range from 1 to 100 nm [21,182]. SPIONs possess interesting magnetic properties and some formulations have already been approved as MRI contrast agents for imaging of the liver by the Food and Drug Administration (FDA) [183]. However, these formulations are no longer available because of concerns about toxicity and fatal anaphylactic reactions. Nowadays, ferumoxytol is the only SPION formulation approved by the FDA for human use, under the commercial name Feraheme^®^. Although its indication is for the treatment of iron deficiency in patients with renal failure, it is also clinically used for MRI of adrenal glands and kidneys [183–184]. As they are composed of iron, a normal component of the human body, SPIONs are believed to have low toxicity and high biocompatibility. SPIONs are metabolized in the lysosomes into a soluble non-superparamagnetic form or iron. Then, iron ions join the iron pool in the blood and can be incorporated by erythrocytes as part of hemoglobin [21,183]. However, the concern about serious anaphylactic reactions, the cause of which is not well understood, seriously restrains their use in clinical treatments. In 2015, the FDA released a “Boxed Warning”, the strongest type of warning, regarding the serious risks following the use of Feraheme. Feraheme can still be used following strict instructions to limit the risk and is under close surveillance by the FDA. Thus, even if SPIONs could be potential tools for imaging the brain and more especially for imaging brain tumors, this issue should be thoroughly investigated.

Similar to AuNPs, SPIONs smaller than 50 nm are able to cross the disrupted BBB around brain tumors or in diseases such as Alzheimer’s or ischemic stroke [185]. Using targeting ligands such as EGF, cetuximab (an anti-EGFR antibody), or anti-Aβ peptide antibodies, their accumulation in these areas can be increased [30,186–187]. For example, in a study by Shevtsov et al., SPIONs conjugated with EGF were tested as contrast agent in an intracranial rat glioma model [30]. Functionalized SPIONs were able to cross the tumor BBB and to accumulate in the tumor and demonstrated high magnetic resonance contrast potential, confirming the ability of functionalized SPIONs to act as a diagnostic agent for intracranial glioma.

## Conclusion

Crossing the BBB remains one of the most challenging tasks for drug delivery to the brain. Different physiological pathways can be employed for crossing the BBB including receptor-mediated transcytosis or adsorptive-mediated transcytosis. To reach this goal, a multitude of nanocarrier systems, such as polymeric, lipid-based or inorganic nanoparticles, have been developed and shown able to cross the BBB owing to their tailored surface properties. In numerous studies, the physical coating of nanoparticles with surfactants and the chemical functionalization with specific ligands have been proven to be successful strategies to enhance the BBB crossing through RMT or AMT. Size and charge of the nanoparticles are also aspects that can influence their penetration into the brain. Smaller nanoparticles are able to cross the BBB more easily and to diffuse better through the brain. However, bigger nanoparticles, if correctly functionalized, are also able to cross the BBB, although to a slightly lower extent. Thus, key to increase the amount of drug delivered to the brain is finding the optimal particle size. Larger particles can be loaded with larger amounts of drugs but will reach the brain in lower concentrations, smaller nanoparticles cannot contain large amounts of API but will reach the brain in higher concentrations. By rationally designing nanoparticle systems, the crossing of the BBB in preclinical studies has been successfully demonstrated repeatedly. However, very few clinical data are available on the efficacy of these strategies in the human body. More efforts should now be taken to accelerate the translation of these results into clinical stages, for instance, by focusing on the behavior of the nanoparticles in human blood and investigating the protein corona forming around them, by investigating the particle–cell interactions or by looking for biomimetic solutions.
